# BRAF Inhibition and UVB Light Synergistically Promote *Mus musculus* Papillomavirus 1-Induced Skin Tumorigenesis

**DOI:** 10.3390/cancers16183133

**Published:** 2024-09-11

**Authors:** Sonja Dorfer, Julia Maria Ressler, Katharina Riebenbauer, Stefanie Kancz, Kim Purkhauser, Victoria Bachmayr, Christophe Cataisson, Reinhard Kirnbauer, Peter Petzelbauer, Markus Wiesmueller, Maximilian Egg, Christoph Hoeller, Alessandra Handisurya

**Affiliations:** 1 Department of Dermatology, Medical University of Vienna, 1090 Vienna, Austria; so.dorfer@crcs.at (S.D.); julia.ressler@meduniwien.ac.at (J.M.R.); kim.purkhauser@meduniwien.ac.at (K.P.); victoria.bachmayr@meduniwien.ac.at (V.B.); reinhard.kirnbauer@meduniwien.ac.at (R.K.); peter.petzelbauer@meduniwien.ac.at (P.P.); markus.wiesmueller@meduniwien.ac.at (M.W.); maximilian.egg@meduniwien.ac.at (M.E.); christoph.hoeller@meduniwien.ac.at (C.H.); 2 Laboratory of Cancer Biology and Genetics, National Cancer Institute, National Institutes of Health, Bethesda, MD 20892, USA; cataissc@mail.nih.gov

**Keywords:** BRAF inhibition, vemurafenib, UVB light, *Mus musculus* papillomavirus 1, MmuPV1, skin tumor

## Abstract

**Simple Summary:**

Outgrowth of skin tumors in cancer patients who receive therapies with BRAF inhibitors is frequently observed as an undesired side effect. Infection with certain human papillomaviruses may play a role in BRAF-inhibitor-associated skin tumor development. In this study, the impact of BRAF inhibitors and Ultraviolet B (UVB) light on tumor growth was investigated in laboratory mice after infection of their skin with a murine papillomavirus. We could show that the combination of BRAF inhibitor treatment and UVB exposure resulted in higher numbers of virus-induced tumors compared with virus-infected mice that had received only BRAF inhibitors, only UVB irradiation or no additional treatment. This shows that BRAF inhibitors, particularly when combined with UVB light, support skin tumor growth in mice and may represent a novel way by which BRAF inhibitors contribute to skin cancer development.

**Abstract:**

The development of keratinocytic skin tumors, presumably attributable to paradoxical activation of the MAPK pathway, represents a relevant side effect of targeted therapies with BRAF inhibitors (BRAFis). The role of cutaneous papillomavirus infection in BRAFi-associated skin carcinogenesis, however, is still inconclusive. Employing the Mus musculus papillomavirus 1 (MmuPV1) skin infection model, the impact of BRAFis and UVB exposure on papillomavirus induced skin tumorigenesis was investigated in immunocompetent FVB/NCrl mice. Systemic BRAF inhibition in combination with UVB light induced skin tumors in 62% of the MmuPV1-infected animals. In contrast, significantly fewer tumors were observed in the absence of either BRAF inhibition, UVB irradiation or virus infection, as demonstrated by lesional outgrowth in 20%, 5% and 0% of the mice, respectively. Combinatory exposure to BRAFis and UVB favored productive viral infection, which was shown by high numbers of MmuPV1 genome copies and *E1^E4* spliced transcripts and an abundance of E6/E7 oncogene mRNA and viral capsid proteins. BRAF inhibition, but not viral infection or UVB light, activated ERK1/2, whereas γH2AX expression, inducible by UVB light, remained unaltered by BRAFis. These results provide experimental evidence that BRAF inhibition and UVB irradiation synergistically promote MmuPV1-induced skin tumor development in vivo. This indicates an alternative pathway by which papillomavirus skin infection may contribute to BRAFi-associated skin tumorigenesis.

## 1. Introduction

Mutations in the proto-oncogene *BRAF* are present in 5–10% of human cancers, including malignant melanoma and colorectal, thyroid and lung cancer [1,2]. In the last few decades, targeted therapies with inhibitors of BRAF (BRAFis), such as vemurafenib, dabrafenib and encorafenib, have emerged as an attractive strategy for oncology patients harboring tumors with *BRAFV600* mutations. For instance, in American Joint Committee on Cancer (AJCC) stage III and IV melanoma patients, combination therapies with BRAFis and MEK inhibitors (MEKis) have become the established standard of care due to their high response rates and improved progression-free and overall survival [3,4].

Despite the high efficacy, unanticipated adverse events, particularly the development of cutaneous squamous cell carcinomas (cSCCs) in 10–37% but also keratoacanthomas and verrucous keratoses in 14–30% and 50–80% of the BRAFi recipients, respectively, remain a concern. Secondary cSCCs develop rapidly after the initiation of BRAFi therapy, with a median time of 8–13 weeks to the first onset [5,6,7,8,9]. The development of single and, less frequently, multiple lesions have been reported, which clinically mostly present as keratoacanthoma-like or wart-like lesions. Affected patients are, on average, above their 60s and rarely below the age of 40 years. The tumors are located predominantly at sun-exposed sites; however, their occurrence at skin sites not exposed to ultraviolet (UV) light has been noted in sporadic cases. Nevertheless, the shorter median latency to cSCC onset on sun-exposed regions compared with sun-protected areas indicates that chronic sun exposure is a contributing risk factor. The reported incidence rates of cSCCs vary between the individual BRAFis and seem lower in the sorafenib and dabrafenib than in the vemurafenib studies [9]. 

The factors driving the formation of BRAFi-associated skin cancers have not been fully elucidated, although, in general, the paradoxical activation of the *RAS/RAF/MEK/ERK* (Mitogen-Activated Protein Kinases, MAPK) pathway by BRAFis is thought to be responsible for tumorigenesis [10]. This paradoxical stimulation is believed to be mediated by the BRAFi-induced dimerization of mutated BRAF and wild-type (WT) RAF isoforms, which enhances cellular responses such as cell proliferation, survival and growth, subsequently driving the progression of skin cancers [11]. Furthermore, upstream activating mutations in *RAS,* particularly in *Hras* (Q61L and G12D) and, to a lesser extent, in *Kras* and *Nras*, were detected in up to 60% of the BRAFi-associated cSCCs, which significantly exceed the rates found in sporadic cSCCs [12,13,14]. These activating mutations may act as an initial stimulus and additionally contribute to the paradoxical MAPK signaling by promoting the dimerization of RAF in cells containing WT BRAF. Finally, BRAFis may initiate perturbation of the auto-inhibited state of WT RAF, thus providing the structural changes needed for RAF dimerization and, ultimately, its paradoxical activation [15]. Although co-administration of BRAFis and MEKis partially counteracted the paradoxical upregulation of the MAPK pathway and, hence, lowered the incidence of secondary skin tumors, complete eradication was not achieved, and cSCCs remained in 1–11% of the patients [16,17]. A substantial proportion of the BRAFi-associated skin tumors, however, cannot be explained by the proposed mechanisms, indicating the involvement of other factors, e.g. genetic, environmental or viral, in skin tumorigenesis.

Cutaneous human papillomaviruses (HPVs) are highly prevalent in the general population; however, certain types, predominantly members of genus beta (beta-HPVs), have oncogenic potential. They cause cSCCs in patients with *Epidermodysplasia verruciformis* and, possibly, in immunocompromised, e.g. organ transplant recipients [18,19], particularly at sun-exposed regions, indicating an adjunct role to the main carcinogen, UV light. In BRAFi-associated skin carcinogenesis, the presence of papillomavirus DNA, koilocytes and p16-positive keratinocytes, the latter two being indicative of HPV infection, in 15–100% of the tumors [9,20,21,22] implicates cutaneous HPV infection as a pathogenetic factor in tumor development. In particular, the beta-HPV types 17, 38 and 111 were among the most frequently detected viral types present in the BRAFi-associated tumors, although viral loads could not be convincingly correlated to the duration of BRAFi treatment [9,23]. Compelling evidence for the functional cooperation between HPVs and BRAFi in promoting cSCC development was provided by several studies involving transgenic (tg) mice expressing the papillomavirus oncogenes [24]. For instance, systemic administration of the BRAFi vemurafenib to K14-HPV16 tg mice markedly increased the incidence of cSCCs when compared with vehicle-treated tg littermates [22]. Similarly, K14-HPV38-E6/E7 tg mice, which express the beta-HPV38 E6 and E7 oncogenes in the basal layer of the epidermis, consistently developed large cSCCs after exposure to BRAF inhibition and UVB light irradiation, whereas equally treated WT controls failed to develop skin tumors [25]. 

Herein, we investigated the impact of BRAF inhibition on *Mus musculus* papillomavirus 1 (MmuPV1)-induced skin tumor development in non-tg, naturally infectable mice to overcome the inherent limitations associated with tg expression of the viral genes. To mimic the sun exposure in an average human, a subset of mice was concomitantly irradiated with defined doses of UVB light, which corresponded approximately to the solar exposure observed in Europe [26,27] and did not cause cSCC development by itself in irradiated animals [25,26,28]. We could demonstrate that the combination of BRAF inhibition together with UVB exposure synergistically promoted papillomavirus-infection-induced skin tumorigenesis. 

## 2. Materials and Methods

### 2.1. Mouse Infection and Treatment Regimens

Animal experiments were approved by the Committee for Animal Experimentation at the Medical University of Vienna, Austria, and the Austrian Federal Ministry of Science and Research (BMWFW−66.009/0304-V/3b/2019) and performed in accordance with the European Convention for Animal Care and Use of Laboratory Animals. 

Immunocompetent FVB/NCrl mice, all females aged 4–5 weeks (Charles River Laboratories, Sulzfeld, Germany), were infected on the skin of the tail with 1 x10^10^ native MmuPV1 virions per site, as previously reported [28,29]. The BRAFi vemurafenib (Zelboraf®, Roche, Basel, Switzerland), dissolved in 0.5% carboxymethylcellulose (CMC) to a dose of 60 mg/kg body weight, or 0.5% CMC as the vehicle control were administered daily via oral gavage in a volume of 0.1 ml to the animals. A subgroup of mice was additionally exposed to UVB irradiation once weekly at 450 mJ/cm^2^ using a Waldmann TP−4 UV lamp (Herbert Waldmann GmbH & Co. KG, Villingen-Schwenningen, Germany), resulting in a cumulative UVB dose of 1800 mJ/cm^2^ (on day 23) or 4050 mJ/cm^2^ (on day 60). The experimental set up is schematically outlined in Supplementary Figure S1. Skin tissues were procured from the inoculation sites at day 23 or 60 post-infection, regardless of whether visible lesions had evolved, and either snap-frozen in liquid nitrogen or fixed in formalin and embedded in paraffin (FFPE) until further analysis.

To facilitate discrimination of the individual treatment regimens, “MmuPV1-infected” mice are hereafter termed “M” mice, “BRAFi-treated” as “B” mice, and “UVB-irradiated” as “U” mice, which were used either alone or in combination, while mice that had not received BRAF inhibition (consisting of vehicle-treated and untreated mice) and mice with no virus, no vehicle and no UV treatment were classified as “Ø”; all groups were subsequently subsumed into the following groups: “MBU”, “MB”, “MU”, “M”, “BU”, “B”, “U” and “Ø” mice.

### 2.2. Immunohistochemistry

Hematoxylin–eosin-stained FFPE skin tissue sections were evaluated by a pathologist blinded to the experimental conditions to verify the presence of tumors. Immunohistochemistry was performed on tissue sections employing the primary antibodies listed in Supplementary Table S1. Images were digitalized using the Aperio slide scanner (Leica Biosystems, Nussloch, Germany). The number of immunopositive cells per mm^2^ tissue and the percentage of immunopositive tissue areas were determined using ImageJ software (Wayne Rasband, USA, https://ij.imjoy.io/) and analyzed as described previously [28].

### 2.3. Western Blotting

Protein lysates were generated from snap-frozen skin tissues by cutting the tissues into very fine pieces. The tissue pieces were subsequently transferred to 2 ml microcentrifuge tubes, each containing a 7 mm stainless steel bead (Qiagen, Venlo, The Netherlands) and phosphate-buffered saline, and homogenized by employing a universal laboratory mixer–mill disruptor (TissueLyser LT; Qiagen) 2 times for 1 min at 50 Hz. After removal of the steel beads from the tubes, the homogenates were subjected to sonication for 2 min at output level 3.0 (Misonix Sonicator 3000 with cup attachment, Misonix Inc., Farmingdale, NY, USA). The suspensions were cleared by centrifugation for 10 min at 5000 × *g* and the supernatants collected. The protein concentrations were measured using the BCA Pierce Protein Assay (Thermo Fisher Scientific, Waltham, MA, USA). Equal amounts of each sample were incubated with 4 x sample buffer (LI-COR Biosciences, Lincoln, NE, USA) for 5 min at 90 °C and separated by 10% SDS-PAGE, and the proteins were subsequently transferred onto a 0.2 µm nitrocellulose membrane. After blocking, the membranes were incubated overnight at 4 °C with primary antibodies recognizing total or phosphorylated (phospho) ERK1/2 or vinculin. Detection was performed with horseradish-peroxidase-labeled secondary antibodies followed by enhanced chemiluminescence- or fluorescence-labeled secondary antibodies and then imaged and analyzed using the Odyssey CLx Infrared Imaging System and Image Studio Version 5.2 (LI-COR Biosciences). The antibodies used are listed in Supplementary Table S1.

### 2.4. Mutation Analyses

Genomic DNA was extracted from FFPE tissue sections using the GeneRead DNA FFPE Kit (Qiagen). For mutation analyses, the DNA was amplified by PCR employing primers specific for mouse *Hras1* codons 12/13 and 61, *Kras* codons 12/13 and 61, and *Nras* codons 12/13 and 61 [30]. The amplicons were purified using the QIAquick PCR Purification Kit (Qiagen) and subjected to Sanger sequencing (Eurofins Genomics, Vienna, Austria). Sequences were obtained from the forward and reverse strands and analyzed using the multiple sequence alignment program Clustal Omega [31].

### 2.5. Quantification of MmuPV1 E1^E4 Splice Transcripts and Viral Copy Numbers 

Total RNA was isolated from snap-frozen skin tissues using the TRI reagent (Sigma-Aldrich, St. Louis, MO, USA) and reverse-transcribed into cDNA by using the High-Capacity cDNA Reverse Transcription Kit (Thermo Fisher Scientific). MmuPV1 *E1^E4* spliced transcripts were determined by real-time PCR using primers specific for *E1^E4* [29]; the results were normalized to the endogenous control, beta-actin (Thermo Fisher Scientific), and the data was analyzed using the comparative CT (ΔΔCT) method [32]. 

To obtain viral copy numbers, qPCR was performed from genomic DNA extracted from the FFPE tissue sections using primers specific for MmuPV1 [29]. The results were normalized to the endogenous control, gamma-actin, and copy numbers were quantified according to standard curves with known amounts of the religated MmuPV1 genome. 

### 2.6. RNA In Situ Hybridization

RNA in situ hybridization was performed on FFPE skin tissue sections employing the RNAscope MusPV-E6-E7 probe (Advanced Cell Diagnostics, Newark, CA, USA), as described previously [28]. Mm-PPIB served as the positive and DapB as the negative control probes. Images were digitalized using the Aperio slide scanner (Leica Biosystems).

### 2.7. Statistics

Data are shown as the mean + standard deviation (SD) of at least three independent experiments. The Student’s *t*-test was applied for the comparison of two groups and one-way analysis of variance (ANOVA) was used for the comparison of more than two groups. *p*-values less than 0.05 were considered statistically significant. Statistical analyses were performed with GraphPad Prism 8 (Version 8.0.2).

## 3. Results

### 3.1. BRAF Inhibition and UVB Irradiation Synergistically Promote Skin Tumor Development in MmuPV1-Infected Mice 

First, the effects of BRAF inhibition on MmuPV1-induced skin tumorigenesis in the presence or absence of UVB light were investigated in FVB/NCrl mice.

**Figure 1:**
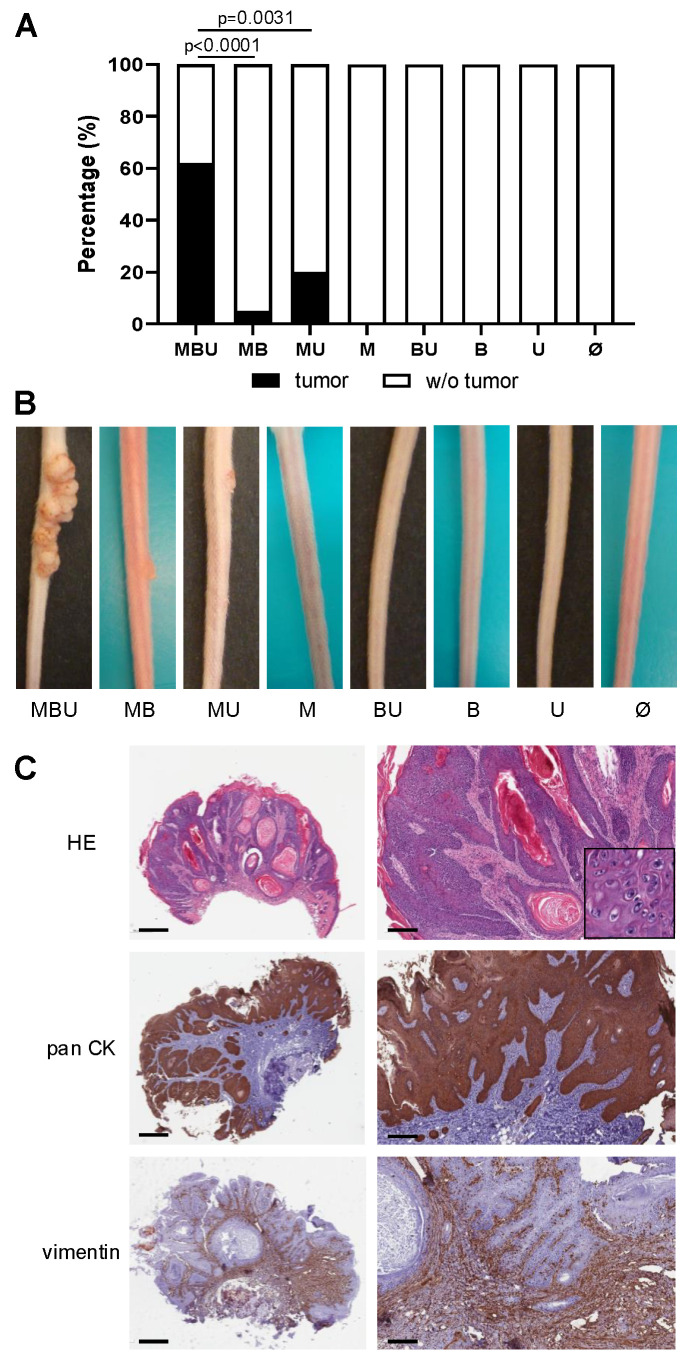
Skin tumor development in immunocompetent FVB/NCrl mice 60 days after infection. (**A**) The bar chart represents the percentage (%) of FVB/NCrl mice with tumors and without (w/o) tumors according to following treatment regimens: MBU, MB, MU, M, BU, B, U, Ø. Statistical significance was determined as *p* ≤ 0.05. (**B**) Exemplary pictures of representative mice and tumor development according to the respective treatment regimen. (**C**) Histological images of a representative skin tumor derived from an MBU mouse. Overview and higher magnification of the same tumors are shown. Top panel: hematoxylin–eosin (HE) staining revealing the typical morphology of cutaneous papillomas with acanthosis, papillomatosis and hyperkeratosis. Insert is of a higher magnification showing the presence of interspersed koilocytes, which corroborates the virus-induced proliferation. Middle panel: pan-cytokeratin (pan CK) staining throughout the tumor tissues demonstrating the epithelial origin of the tumorous cells. Lower panel: vimentin staining predominantly in the underlying supporting connective tissues and in a few epithelial cells. Scale bars represent 700 µm (left side, overview) and 200 µm (right side).

Strikingly, tumor development occurred in the majority (62%; 8/13) of MBU mice (Figure 1A,B). Lesion outgrowth started at around day 16 after viral skin infection, and by day 60, the tumors had an average length and height of 8.2 (±5.7) mm and 1.5 (±0.8) mm, respectively. In contrast, in the absence of one component, namely systemic BRAF inhibition or UVB light, significantly fewer virus-induced skin tumors were observed. Tumor outgrowth was noted in 20% (3/15) of the MU mice, which had not received the BRAFi, and the lesions were smaller, with an average length of 3.8 (±0.4) mm and 0.4 (±0.3) mm tumor thickness on day 60 post-infection. Without UVB irradiation, a tumor was found in 5% (1/20) of the MB mice, which was significantly lower compared with MBU mice albeit indicative of a modest supportive effect of BRAF inhibition on virus-induced tumorigenesis. Skin tumor development was strictly dependent upon MmuPV1 infection, as lesions were not observed in uninfected mice over the entire study period (0%; 0/70), regardless of treatment with BRAFi (B mice: 0%; 0/20), UVB light (U mice: 0%; 0/15) or a combination of both (BU mice: 0%; 0/15). Further, the tail epidermal thickness of BU and U mice was relatively similar on day 60 at 28.7 (±3.7) μm and 31.5 (±7.5) μm, respectively. Notably, virus infection alone did not lead to tumor formation (M mice 0%; 0/20).

Histologically, the abundant expression of pan-cytokeratin throughout the tumor tissues confirmed the epithelial origin of the cells (Figure 1C).

### 3.2. BRAF Inhibition, but Not MmuPV1 Infection, Activates ERK1/2 Signaling 

To investigate whether the tumor outgrowth in our mice was attributable to BRAFi-induced paradoxical activation of MAPK signaling and whether MAPK activation could be potentiated by MmuPV1 infection and UVB light, the levels of downstream phospho and total ERK1/2 were determined in the skin tissues.

**Figure 2:**
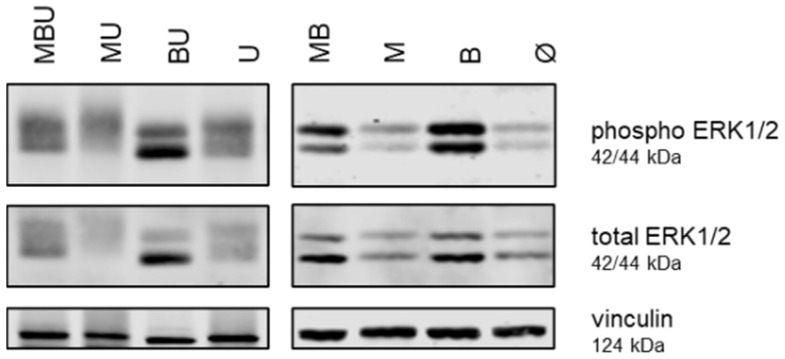
Expression of phosphorylated (phospho) and total ERK1/2 in murine skin tissues. Western blot analysis of phospho ERK1/2, total ERK1/2 and vinculin expression in murine skin tissues derived from representative MBU, MB, MU, M, BU, B, U and Ø mice. The respective molecular weights are indicated for each protein.

Overall, the levels of phospho and total ERK1/2 were higher in the tissues derived from mice in which BRAF had been systemically inhibited compared with tissues derived from mice which had not received the BRAFi (Figure 2). Otherwise, MmuPV1 infection as well as UVB irradiation had no major effect on ERK1/2 phosphorylation in the skin tissues.

### 3.3. Mutations in Hras1, Kras and Nras Are Absent in the Murine Skin Tumors

To determine whether activating mutations in *RAS* could have contributed to tumor development in our mice, either via BRAFi-induced MAPK-dependent [14] or MAPK-independent [33,34] initiation and promotion of tumorigenesis, tumor tissues were analyzed for mutations in *Hras1, Kras* and *Nras*, all in the hotspot codons 12, 13 and 61. In the tumors derived from MBU mice, mutations were neither detectable in *Hras1* codons 12/13 nor in codon 61 (both 0%; each 0/5). Similarly, in the *Kras* codons 12/13 and 61 and the *Nras* codons 12/13 and 61, no mutations were identified in the tumor tissues of MBU mice (for each isoform and codon 0%; 0/5). In the absence of BRAF inhibition, the combination of MmuPV1 infection and concomitant UVB irradiation did not result in mutations in codons 12/13 and 61 of *Hras1, Kras* and *Nras* (MU mice: all 0%; 0/4 each), and UVB irradiation alone likewise did not induce mutations in any of the investigated *RAS* isoforms (U mice: all 0%; 0/1 each).

### 3.4. BRAF Inhibition and UVB Light Synergistically Favor Papillomavirus Infection

As the high incidence in our MBU mice could neither be attributed to increased phospho ERK1/2 levels or underlying activating mutations in the investigated *RAS* isoforms, next, we evaluated the impact of BRAF inhibition in the context of UVB irradiation on papillomavirus infection.

**Figure 3:**
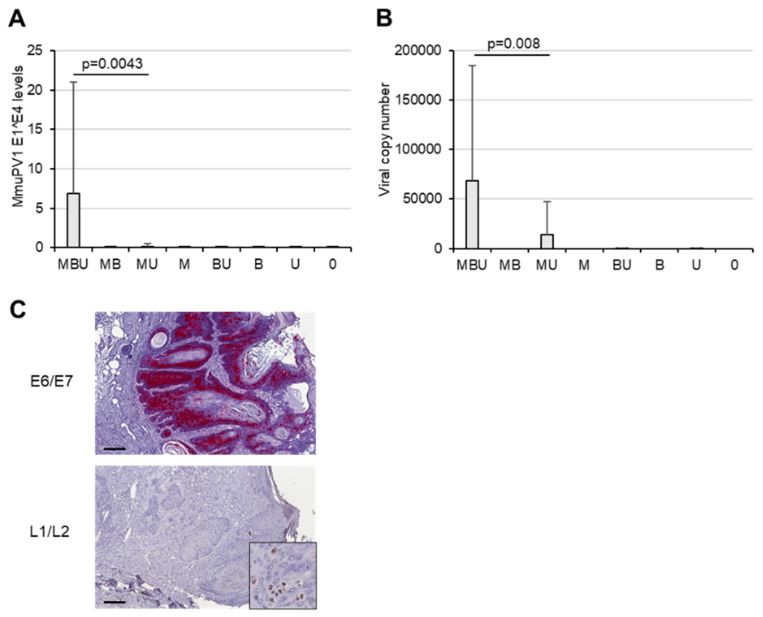
Effect of BRAF inhibition and UVB light on papillomavirus infection. (**A**) MmuPV1-*E1^E4* spliced transcripts in skin tissues according to the individual treatment regimen (statistical significance determined by *p* ≤ 0.05). (**B**) MmuPV1 genome copy numbers in skin tissues of mice, according to treatment. (**C**) Top panel: MmuPV1-E6/E7 mRNA in a skin tumor of a representative MBU mouse, as determined by RNAscope. Lower panel: immunohistochemical staining for MmuPV1-L1/L2 capsid proteins present in the tumor of a MBU mouse. Insert shows a higher magnification. Scale bars indicate 200 µm.

BRAFi plus UVB irradiation increased the levels of MmuPV1 *E1^E4* spliced transcripts, which represent a marker of active viral infection (Figure 3A). The *E1^E4* levels were highest in MBU mice and 44-fold higher when compared with MU mice, which had not received systemic BRAF inhibition. Viral *E1^E4* transcripts were almost undetectable in MB and M mice and, as expected, absent in uninfected mice. Similarly, the highest MmuPV1 genome copy numbers were observed in the tissues derived from MBU mice (Figure 3B). The copy numbers were 5-fold higher compared with MU mice and undetectable in all other mice. In accordance with these results, the tumors of MBU mice harbored abundant amounts of the viral E6/E7 oncogene mRNA throughout the entire epithelium (Figure 3C), demonstrating strong transcriptional activity. Furthermore, detection of the viral capsid proteins in the tumor cells of MBU mice revealed the presence of structural viral proteins (Figure 3C), which provide the platform for virion assembly and the subsequent productive viral infection.

### 3.5. MmuPV1 Infection and UVB Light, but Not BRAF Inhibition, Affect Genomic Stability 

Next, we investigated the role of genomic damage in the development of the skin tumors by employing γH2AX as a functional surrogate marker for DNA double-stranded breaks and chromatin instability. 

**Figure 4:**
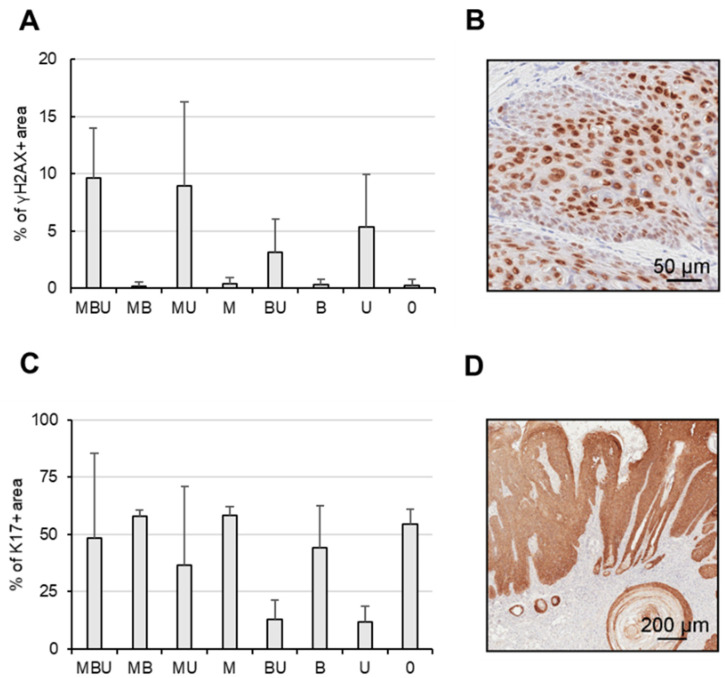
Expression of γH2AX and stress keratin 17 in the murine tissues. (**A**) γH2AX expression in the skin tissues stratified according to treatment regimen. Skin tissues were harvested within 24 h after the final UVB irradiation. (**B**) Representative tissue derived from a MBU mouse stained for γH2AX. (**C**) Keratin 17 expression in the murine skin tissues according to treatment regimen. (**D**) Representative tissue derived from a tumor-bearing MBU mouse stained for keratin 17.

Overall, the expression of γH2AX was strongly dependent upon exposure to UVB light and, to a lesser extent, on MmuPV1 infection (Figure 4A). While the highest levels were detected in tissues derived from MBU, they did not differ significantly from the levels observed in tissues obtained from MU, BU or U mice, with a variation of less than 3.2-fold. Inhibition of BRAF had no major effect on γH2AX expression, as shown by the relatively comparable levels in MBU versus MU, BU versus U or MB versus M mice, all of which showed a difference of less than 1.2-fold in their immunopositivity.

### 3.6. Stress Keratin 17 (K17) Expression Is Linked to Skin Tumor Outgrowth in MBU Mice

To address whether tumor development in our mice was associated with suppression of the host´s cellular immune responses via overexpression of stress keratins, which, in turn, enhances papillomavirus-associated disease, as recently reported [35], we investigated the expression of K17 in the tissues.

Surprisingly, K17 expression was observed in all skin tissues (Figure 4B). Systemic BRAFi treatment did not significantly alter K17 expression in either virus-infected or uninfected mice. Similarly, MmuPV1 infection had no significant effect on K17 levels. Contrary, UVB exposure, in general, reduced K17 in the range of 3–8-fold, as demonstrated, for instance, in M versus MU, B versus BU or Ø versus U mice. However, overall, in MBU mice, K17 expression was relatively high, despite the exposure to UVB. To address whether K17 expression differs between tumor-bearing and non-tumor-bearing mice, the tissues of the MBU mice were analyzed individually according to the presence of tumors. In the tumors, 80.1% immunopositivity for K17 was observed, which is in contrast to 16.7% in the corresponding non-tumorous animals.

## 4. Discussion

In this study, we provide experimental evidence that BRAF inhibition and UVB irradiation cooperate in skin tumorigenesis in a natural papillomavirus-infection-based mouse model. Although the paradoxical activation of the MAPK pathway through amplification of upstream MAPK components remains the main mechanism for secondary skin tumor development in BRAFi recipients, our study indicate that other additional factors may have a contributory effect in a subset of the BRAFi-associated tumors. In particular, it is conceivable that BRAF inhibition in conjunction with UVB irradiation may utilize properties of the papillomavirus to contribute to skin tumorigenesis and, possibly, ultimately to skin cancer. 

This may particularly apply to those special cases in which activating mutations in *RAS* are absent (such as observed in more than 40% of human BRAFi-associated cSCCs [12,13,14]) or when tumor development occurs independently of MAPK activation [33,34]. In this regard, this scenario may be reflected by our MBU mice, in which significantly more tumors were observed. The higher incidences were not attributable to viral infection and UVB light alone, although the development of skin tumors, including cSCCs, induced by MmuPV1 and UVB were reported previously [36]. In this experimental system [28], lower UVB doses, imitating the average annual exposure of a human individual in Europe [27], and shorter exposure periods were employed that did not suffice to induce the severe immunosuppression necessary for consistent tumor outgrowth [25]. Notably, FVB/NCrl mice do not harbor *BRAFV600* mutations but, rather, WT BRAF; hence, one could assume that the BRAFi should not exert any specific effect due to the lack of a specific target. However, vemurafenib/PLX4032 was previously shown to be capable of inducing MEK and ERK phosphorylation in a variety of cells with WT BRAF [37], suggesting that BRAFi may still influence MAPK activation in the absence of mutated *RAF*. However, the presence of the BRAFi alone in the absence of both co-factors, MmuPV1 infection and UVB light, did not result in sufficient cell proliferation to consistently generate hyperplasia or tumors. Therefore, we further investigated how the combination of all three factors—BRAF inhibition, UVB light and papillomaviral infection (in the absence of mutated BRAF and *RAS* activating mutations)—supports skin carcinogenesis and, more specifically, if the presence of the BRAFi in conjunction with UVB, could have an effect on MmuPV1 infection in inducing skin tumorigenesis. In line with this, MmuPV1 was previously shown to have carcinogenic potential in causing the outgrowth of large cSCCs in severely cyclosporin A-treated mice, even in the absence of any UVB light [28]. 

Beta-HPVs are thought to be acquired early in life in the general population and can already be detected in the skin of infants and young children, most likely through direct contact with the skin of the parents. Due to the almost ubiquitous spread and general lack of symptoms, cutaneous beta-HPVs are thought to have the ability to persist as subclinical productive infections, with a reservoir for latent infection being the hair follicle stem cells [38]. Prevalence increases with age, cumulative sun exposure and, particularly, after immunosuppression, indicating that papillomavirus activity is controlled by the host´s normal immune-surveillance mechanisms and that an imbalance of immune control, occurring under prolonged UV or iatrogenic immunosuppression or possibly also under BRAFi therapy, may allow for reactivation of the virus or the establishment of a profound viral infection. This kind of virus latency is observed, for instance, in members of the Herpes virus family, such as the Herpes simplex and Epstein Barr viruses and cytomegalovirus.

The increase in and persistence of papillomaviral markers in the MBU mice, contrary to infected controls, are striking. The papillomaviral *E1^E4* spliced transcripts, for instance, have been shown to favor persistence of the virus, to promote viral genome amplification and to prevent viral clearance [39]. It is conceivable that inhibition of BRAF in the context of locally immunosuppressive UVB light [40] may directly allow for or support the establishment of a profound viral infection and, thus, promote persistence of the virus in skin tissues that would otherwise have been cleared by the murine immune system. The prolonged viral E6/E7 oncogene expression in the tissues, in turn, could alter the promotion threshold for virally driven cells, allowing for their expansion. Furthermore, upregulation of MAPK signaling per se has been shown to enhance papillomavirus replication, stability and infectivity [39,41]. Hence, it is also possible that the BRAFi-induced interference in our MBU mice, while moderate, may have indirectly supported viral infection and persistence or impaired the elimination of an oncogenic papillomavirus via the activation of ERK1/2. Contrary to high-risk mucosal HPV-induced anogenital (particularly cervical) cancer, in cutaneous HPV-induced skin carcinogenesis, a “hit-and-run” mechanism is generally proposed, which involves impaired repair of UV-induced DNA damage or the prevention of apoptosis of damaged cells [42,43]. In this regard, expression of γH2AX, which is considered a biomarker of genomic instability, was elevated in the skin of MBU mice. Hence, taken together, the inhibition of BRAF, more efficiently in combination with UVB light, may constitute a favorable milieu for the persistence and/or reactivation of a latent papillomaviral infection, which allows for skin tumor development similarly to that following chronic immunosuppression in organ transplant patients and as shown previously in cyclosporin A-immunosuppressed MmuPV1-infected mice [28,44]. While the exact pathways by which cutaneous HPVs induce skin carcinogenesis have not yet completely been elucidated [18,19,42,43], the presence of cutaneous papillomaviruses may account for the special cases of secondary cSCCs that cannot be attributed to the classical paradoxical MAPK activation. 

Organ-transplanted patients have benefited from widespread immunization efforts prior to transplantation [45]. The current licensed HPV vaccines are efficacious and established as a strategy to prevent mucosal HPV-induced diseases, including dysplasia and cancer of the anogenital and the oropharyngeal tract. While no HPV vaccine against cutaneous types is available at the moment, the proof of concept for the feasibility of cutaneous HPV vaccination in preventing HPV-induced skin diseases exists [46,47,48], and several new broad-spectrum HPV vaccine candidates, mostly based on the virus minor capsid protein L2, are currently being evaluated [49,50]. These vaccines were shown to induce antibodies that cross-neutralized non-cognate cutaneous types in vitro, and, importantly, cross-neutralization was also achieved in vivo after cutaneous challenge with papillomavirus-based pseudovirions. This demonstrates that cutaneous HPV vaccination may become a future tool capable of protecting against papillomaviral skin diseases, including, possibly, keratinocytic tumors.

K17 represents an intermediate filament-forming cytoskeletal protein, and under physiological conditions, it is not constitutively expressed, but it is induced in transformed epithelial tissues [51]. It can modulate the host cellular immune response and is required for efficient and continued tumor growth in immunocompetent mice. In MmuPV1-infected mice, expression of K17 was previously reported to be required for efficient and continued papilloma outgrowth and for the amplification of diseases related to papillomavirus infection by downregulating T-cell recruitment [35]. In line with the previous report, we observed that elevated K17 levels were associated with virus-induced tumor outgrowth in our mice and detected lower CD8^+^ and CD4^+^ T-cell numbers in MBU compared with, for instance, M mice (our preliminary results). Future in-depth investigations on whether and, if so, how T-cells may contribute to tumorigenesis in human or murine BRAFi recipients are warranted.

Contrary to previous reports on BRAFi-associated cSCCs in which somatic mutations in *RAS* were identified in 18–60% [12,13,14], in our murine tumors, activating mutations in the hotspot regions of *Hras1, Kras* and *Nras*, which might have contributed to tumor development, were not detected. However, in our tumors, we cannot exclude the presence of secondary mutations, e.g., in *BRAF* or *MEK1* per se or in other cancer-associated proteins, such as *PIK3CA, CKIT, ALK* and *EGFR* (as reported for BRAFi-associated cSCCs [9], or the presence of UV-induced signature mutations in genes such as *RAC1*, *p53* and *PTEN*. Furthermore, herein, we investigated whole tumorous tissues, which consist of different cell types instead of single tumor cells. It is possible that mutations present in some tumor cells might have been masked by the bulk of WT *RAS* present in other cell types, or in case where there are less than 25% of such cells, the mutant DNA might not have been detected [52]. Another limitation of the study is the treatment period of 60 days for the BRAFi, which allowed for the assessment of the development of benign virus-induced tumors but not of malignant progression and cSCCs, which generally occur after a period of 30 weeks in severely immunosuppressed mice [26,28]. However, daily oral gavage of BRAFi is tedious and causes a lot of stress for the mice, which, hence, limited the experimental period. Nevertheless, oral gavage was chosen to ensure that each mouse received the same amount of BRAFi according to its body weight and to rule out any possible feeding advantage of a dominant mouse within the group. In future studies, the treatment period could be prolonged, the BRAFi could be administered orally without gavage and the cumulative UV dose could be increased to expand our model to allow for investigations on cSCC development. Furthermore, the model can be adapted to evaluate skin tumor occurrence in the presence of a combination therapy with BRAFis and MEKis, since this combination therapy is increasingly being used; for instance, in the adjuvant setting of stage III melanoma [53], with an expected expansion to stage II melanoma in the future. This is of clinical relevance, because BRAFis/MEKis and checkpoint inhibitors have similar activity in stage III melanoma in the adjuvant setting [54], and it is therefore crucial to identify and mitigate risk factors in order to provide each patient with the most suitable treatment modality where possible.

## 5. Conclusions

In summary, this study provides experimental evidence that inhibition of BRAF and UVB light act synergistically in favoring papillomavirus-infection-induced skin tumor development in vivo, and it reveals a novel pathway additional to the paradoxical activation of MAPK signaling. Future investigations are warranted, given the increasing use of BRAFis/MEKis in the adjuvant treatment of early-stage melanoma and of BRAFis in other tumor entities, such as colon cancer, non-small-cell lung cancer, thyroid cancer and pediatric glioblastoma, to ultimately provide adequate preventive strategies to protect recipients of BRAFis and possibly other targeted therapies from undesirable secondary cancers. 

## Data Availability

The datasets generated and analyzed in the current study are available from the corresponding author upon reasonable request.

## Supplementary Materials

The following supporting information can be downloaded at: https://www.mdpi.com/article/10.3390/cancers16183133/s1, Figure S1: Experimental set up of the in vivo experiments, Figure S2: Uncropped Western blot of Figure 2; Table S1: List of antibodies used for Western blotting and immunohistochemistry.

## Abbreviations

The following abbreviations are used in this manuscript:

BRAFi	BRAF inhibitor
AJCC	American Joint Committee on Cancer
MEKi	Mitogen-activated protein inhibitor
cSCCs	Cutaneous squamous cell carcinomas
UV	Ultraviolet light
MAPK	Mitogen-activated protein kinase
WT	Wild-type
HPV	Human papillomavirus
tg	Transgenic
MmuPV1	*Mus musculus* papillomavirus 1
CMC	Carboxymethyl cellulose
FFPE	Formalin-fixed and paraffin-embedded
M	MmuPV1-infected mice
B	BRAF-inhibitor-treated mice
U	UVB-irradiated mice
Ø	Vehicle-treated mice
MBU	MmuPV1-infected, BRAFi-treated, UVB-irradiated mice
MB	MmuPV1-infected, BRAFi-treated mice
MU	MmuPV1-infected, UVB-irradiated mice
BU	BRAF-inhibitor-treated and UVB-irradiated mice
DMSO	Dimethyl sulfoxide
K17	Keratin 17

## References

[1] Hertzman Johansson C., Egyhazi Brage S. (2014). BRAF inhibitors in cancer therapy. Pharmacol. Ther..

[2] Pisapia P., Pepe F., Iaccarino A., Sgariglia R., Nacchio M., Russo G., Gragnano G., Malapelle U., Troncone G. (2020). BRAF: A Two-Faced Janus. Cells.

[3] Robert C., Grob J.J., Stroyakovskiy D., Karaszewska B., Hauschild A., Levchenko E., Chiarion Sileni V., Schachter J., Garbe C., Bondarenko I. (2019). Five-Year Outcomes with Dabrafenib plus Trametinib in Metastatic Melanoma. New Engl. J. Med..

[4] Chapman P.B., Hauschild A., Robert C., Haanen J.B., Ascierto P., Larkin J., Dummer R., Garbe C., Testori A., Maio M. (2011). Improved survival with vemurafenib in melanoma with BRAF V600E mutation. New Engl. J. Med..

[5] Anforth R., Menzies A., Byth K., Carlos G., Chou S., Sharma R., Scolyer R.A., Kefford R., Long G.V., Fernandez-Peñas P. (2015). Factors influencing the development of cutaneous squamous cell carcinoma in patients on BRAF inhibitor therapy. J. Am. Acad. Dermatol..

[6] Boussemart L., Routier E., Mateus C., Opletalova K., Sebille G., Kamsu-Kom N., Thomas M., Vagner S., Favre M., Tomasic G. (2013). Prospective study of cutaneous side-effects associated with the BRAF inhibitor vemurafenib: A study of 42 patients. Ann. Oncol..

[7] Lacouture M.E., Duvic M., Hauschild A., Prieto V.G., Robert C., Schadendorf D., Kim C.C., McCormack C.J., Myskowski P.L., Spleiss O. (2013). Analysis of dermatologic events in vemurafenib-treated patients with melanoma. Oncologist.

[8] Mattei P.L., Alora-Palli M.B., Kraft S., Lawrence D.P., Flaherty K.T., Kimball A.B. (2013). Cutaneous effects of BRAF inhibitor therapy: A case series. Ann. Oncol..

[9] Cohen D.N., Lawson S.K., Shaver A.C., Du L., Nguyen H.P., He Q., Johnson D.B., Lumbang W.A., Moody B.R., Prescott J.L. (2015). Contribution of Beta-HPV Infection and UV Damage to Rapid-Onset Cutaneous Squamous Cell Carcinoma during BRAF-Inhibition Therapy. Clin. Cancer Res..

[10] Pérez-Lorenzo R., Zheng B. (2012). Targeted inhibition of BRAF kinase: Opportunities and challenges for therapeutics in melanoma. Biosci. Rep..

[11] Lavoie H., Gagnon J., Therrien M. (2020). ERK signalling: A master regulator of cell behaviour, life and fate. Nat. Rev. Mol. Cell. Biol..

[12] Anforth R., Tembe V., Blumetti T., Fernandez-Peñas P. (2012). Mutational analysis of cutaneous squamous cell carcinomas and verrucal keratosis in patients taking BRAF inhibitors. Pigment Cell Melanoma Res..

[13] Oberholzer P.A., Kee D., Dziunycz P., Sucker A., Kamsukom N., Jones R., Roden C., Chalk C.J., Ardlie K., Palescandolo E. (2012). RAS mutations are associated with the development of cutaneous squamous cell tumors in patients treated with RAF inhibitors. J. Clin. Oncol..

[14] Su F., Viros A., Milagre C., Trunzer K., Bollag G., Spleiss O., Reis-Filho J.S., Kong X., Koya R.C., Flaherty K.T. (2012). RAS mutations in cutaneous squamous-cell carcinomas in patients treated with BRAF inhibitors. New Engl. J. Med..

[15] Holderfield M., Merritt H., Chan J., Wallroth M., Tandeske L., Zhai H., Tellew J., Hardy S., Hekmat-Nejad M., Stuart D.D. (2013). RAF inhibitors activate the MAPK pathway by relieving inhibitory autophosphorylation. Cancer Cell.

[16] Larkin J., Ascierto P.A., Dréno B., Atkinson V., Liszkay G., Maio M., Mandalà M., Demidov L., Stroyakovskiy D., Thomas L. (2014). Combined vemurafenib and cobimetinib in BRAF-mutated melanoma. New Engl. J. Med..

[17] Long G.V., Stroyakovskiy D., Gogas H., Levchenko E., de Braud F., Larkin J., Garbe C., Jouary T., Hauschild A., Grob J.J. (2014). Combined BRAF and MEK inhibition versus BRAF inhibition alone in melanoma. New Engl. J. Med..

[18] Howley P.M., Pfister H.J. (2015). Beta genus papillomaviruses and skin cancer. Virology.

[19] Quint K.D., Genders R.E., de Koning M.N., Borgogna C., Gariglio M., Bouwes Bavinck J.N., Doorbar J., Feltkamp M.C. (2015). Human Beta-papillomavirus infection and keratinocyte carcinomas. J. Pathol..

[20] Falchook G.S., Rady P., Konopinski J.C., Busaidy N., Hess K., Hymes S., Nguyen H.P., Prieto V.G., Bustinza-Linares E., Lin Q. (2016). Merkel cell polyomavirus and human papilloma virus in proliferative skin lesions arising in patients treated with BRAF inhibitors. Arch. Dermatol. Res..

[21] Schrama D., Groesser L., Ugurel S., Hafner C., Pastrana D.V., Buck C.B., Cerroni L., Theiler A., Becker J.C. (2014). Presence of human polyomavirus 6 in mutation-specific BRAF inhibitor-induced epithelial proliferations. JAMA Dermatol..

[22] Holderfield M., Lorenzana E., Weisburd B., Lomovasky L., Boussemart L., Lacroix L., Tomasic G., Favre M., Vagner S., Robert C. (2014). Vemurafenib cooperates with HPV to promote initiation of cutaneous tumors. Cancer Res..

[23] Purdie K.J., Proby C.M., Rizvi H., Griffin H., Doorbar J., Sommerlad M., Feltkamp M.C., der Meijden E.V., Inman G.J., South A.P. (2018). The Role of Human Papillomaviruses and Polyomaviruses in BRAF-Inhibitor Induced Cutaneous Squamous Cell Carcinoma and Benign Squamoproliferative Lesions. Front. Microbiol..

[24] Wu J.H., Cohen D.N., Rady P.L., Tyring S.K. (2017). BRAF inhibitor-associated cutaneous squamous cell carcinoma: New mechanistic insight, emerging evidence for viral involvement and perspectives on clinical management. Br. J. Dermatol..

[25] Viarisio D., Müller-Decker K., Hassel J.C., Alvarez J.C., Flechtenmacher C., Pawlita M., Gissmann L., Tommasino M. (2017). The BRAF Inhibitor Vemurafenib Enhances UV-Induced Skin Carcinogenesis in Beta HPV38 E6 and E7 Transgenic Mice. J. Invest. Dermatol..

[26] Viarisio D., Müller-Decker K., Accardi R., Robitaille A., Dürst M., Beer K., Jansen L., Flechtenmacher C., Bozza M., Harbottle R. (2018). Beta HPV38 oncoproteins act with a hit-and-run mechanism in ultraviolet radiation-induced skin carcinogenesis in mice. PLoS Pathog..

[27] Khanna T., Shraim R., Zarkovic M., van Weele M., van Geffen J., Zgaga L. (2022). Comprehensive Analysis of Seasonal and Geographical Variation in UVB Radiation Relevant for Vitamin D Production in Europe. Nutrients.

[28] Dorfer S., Strasser K., Schröckenfuchs G., Bonelli M., Bauer W., Kittler H., Cataisson C., Fischer M.B., Lichtenberger B.M., Handisurya A. (2021). Mus musculus papillomavirus 1 is a key driver of skin cancer development upon immunosuppression. Am. J. Transplant..

[29] Handisurya A., Day P.M., Thompson C.D., Buck C.B., Pang Y.Y., Lowy D.R., Schiller J.T. (2013). Characterization of Mus musculus papillomavirus 1 infection in situ reveals an unusual pattern of late gene expression and capsid protein localization. J. Virol..

[30] De Pedro I., Alonso-Lecue P., Sanz-Gómez N., Freije A., Gandarillas A. (2018). Sublethal UV irradiation induces squamous differentiation via a p53-independent, DNA damage-mitosis checkpoint. Cell Death Dis..

[31] Cataisson C., Michalowski A.M., Shibuya K., Ryscavage A., Klosterman M., Wright L., Dubois W., Liu F., Zhuang A., Rodrigues K.B. (2016). MET signaling in keratinocytes activates EGFR and initiates squamous carcinogenesis. Sci. Signal..

[32] Madeira F., Pearce M., Tivey A.R.N., Basutkar P., Lee J., Edbali O., Madhusoodanan N., Kolesnikov A., Lopez R. (2022). Search and sequence analysis tools services from EMBL-EBI in 2022. Nucleic Acids Res..

[33] Livak K.J., Schmittgen T.D. (2001). Analysis of relative gene expression data using real-time quantitative PCR and the 2^−ΔΔ*C*^_T_ Method. Methods.

[34] Cataisson C., Lee A.J., Zhang A.M., Mizes A., Korkmaz S., Carofino B.L., Meyer T.J., Michalowski A.M., Li L., Yuspa S.H. (2022). RAS oncogene signal strength regulates matrisomal gene expression and tumorigenicity of mouse keratinocytes. Carcinogenesis.

[35] Dellambra E. (2016). Oncogenic Ras: A double-edged sword for human epidermal stem and transient amplifying cells. Small GTPases.

[36] Wang W., Uberoi A., Spurgeon M., Gronski E., Majerciak V., Lobanov A., Hayes M., Loke A., Zheng Z.M., Lambert P.F. (2020). Stress keratin 17 enhances papillomavirus infection-induced disease by downregulating T cell recruitment. PLoS Pathog..

[37] Uberoi A., Yoshida S., Frazer I.H., Pitot H.C., Lambert P.F. (2016). Role of Ultraviolet Radiation in Papillomavirus-Induced Disease. PLoS Pathog..

[38] Gheit T. (2019). Mucosal and Cutaneous Human Papillomavirus Infections and Cancer Biology. Front. Oncol..

[39] Wang Q., Kennedy A., Das P., McIntosh P.B., Howell S.A., Isaacson E.R., Hinz S.A., Davy C., Doorbar J. (2009). Phosphorylation of the human papillomavirus type 16 E1^E4 protein at T57 by ERK triggers a structural change that enhances keratin binding and protein stability. J. Virol..

[40] Di Nuzzo S., Sylva-Steenland R.M., de Rie M.A., Das P.K., Bos J.D., Teunissen M.B. (1998). UVB radiation preferentially induces recruitment of memory CD4^+^ T cells in normal human skin: Long-term effect after a single exposure. J. Invest. Dermatol..

[41] Bowser B.S., Alam S., Meyers C. (2011). Treatment of a human papillomavirus type 31b-positive cell line with benzo[a]pyrene increases viral titer through activation of the Erk1/2 signaling pathway. J. Virol..

[42] Lanz J., Bouwes Bavinck J.N., Westhuis M., Quint K.D., Harwood C.A., Nasir S., Van-de-Velde V., Proby C.M., Ferrándiz C., Genders R.E. (2019). Aggressive Squamous Cell Carcinoma in Organ Transplant Recipients. JAMA Dermatol..

[43] Hasche D., Akgül B. (2022). Role of human papillomavirus (HPV) in the development of skin cancer. Hautarzt.

[44] Bandolin L., Borsetto D., Fussey J., Da Mosto M.C., Nicolai P., Menegaldo A., Calabrese L., Tommasino M., Boscolo-Rizzo P. (2020). Beta human papillomaviruses infection and skin carcinogenesis. Rev. Med. Virol..

[45] Kitano T., Schwartz K.L., Abdulnoor M., Garfield H., Booran N.K., Avitzur Y., Teoh C.W., Hébert D., Allen U. (2023). Immunogenicity of a quadrivalent human papillomavirus vaccine in pediatric kidney and liver transplant recipients. Pediatr. Transplant..

[46] Senger T., Schädlich L., Textor S., Klein C., Michael K.M., Buck C.B., Gissmann L. (2010). Virus-like particles and capsomeres are potent vaccines against cutaneous alpha HPVs. Vaccine.

[47] Vinzón S.E., Braspenning-Wesch I., Müller M., Geissler E.K., Nindl I., Gröne H.J., Schäfer K., Rösl F. (2014). Protective vaccination against papillomavirus-induced skin tumors under immunocompetent and immunosuppressive conditions: A preclinical study using a natural outbred animal model. PLoS Pathog..

[48] Vinzón S.E., Rösl F. (2015). HPV vaccination for prevention of skin cancer. Hum. Vaccin. Immunother..

[49] Ahmels M., Mariz F.C., Braspenning-Wesch I., Stephan S., Huber B., Schmidt G., Cao R., Müller M., Kirnbauer R., Rösl F. (2022). Next generation L2-based HPV vaccines cross-protect against cutaneous papillomavirus infection and tumor development. Front. Immunol..

[50] Mariz F.C., Balz K., Dittrich M., Zhang Y., Yang F., Zhao X., Bolchi A., Ottonello S., Müller M. (2022). A broadly protective vaccine against cutaneous human papillomaviruses. NPJ Vaccines.

[51] Chiang C.H., Wu C.C., Lee L.Y., Li Y.C., Liu H.P., Hsu C.W., Lu Y.C., Chang J.T., Cheng A.J. (2016). Proteomics Analysis Reveals Involvement of Krt17 in Areca Nut-Induced Oral Carcinogenesis. J. Proteome Res..

[52] Peris K., Fargnoli M.C., Garbe C., Kaufmann R., Bastholt L., Seguin N.B., Bataille V., Marmol V.D., Dummer R., Harwood C.A. (2019). Diagnosis and treatment of basal cell carcinoma: European consensus-based interdisciplinary guidelines. Eur. J. Cancer.

[53] Long G.V., Hauschild A., Santinami M., Kirkwood J.M., Atkinson V., Mandala M., Merelli B., Sileni V.C., Nyakas M., Haydon A. (2024). Final Results for Adjuvant Dabrafenib plus Trametinib in Stage III Melanoma. New Engl. J. Med..

[54] Schumann K., Mauch C., Klespe K.C., Loquai C., Nikfarjam U., Schlaak M., Akçetin L., Kölblinger P., Hoellwerth M., Meissner M. (2023). Real-world outcomes using PD-1 antibodies and BRAF +  MEK inhibitors for adjuvant melanoma treatment from 39 skin cancer centers in Germany, Austria and Switzerland. J. Eur. Acad. Dermatol. Venereol..

