# Supplementary material for: BRAF Inhibition and UVB Light Synergistically Promote Mus musculus Papillomavirus 1-Induced Skin Tumorigenesis
Source: Cancers (Basel). 2024 Sep 11;16(18):3133. doi: 10.3390/cancers16183133 (PMC11440113; doi:10.3390/cancers16183133)
Supplement: Supplementary file 1 [file cancers-16-03133-s001.zip › cancers-3172833-supplementary.pdf]

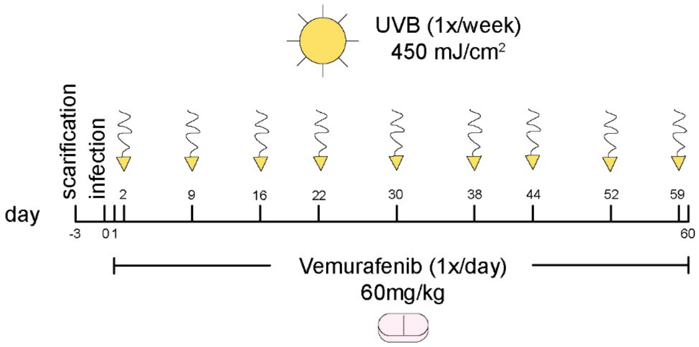

Supplementary Figure S1. Experimental set up of the in vivo experiments.

Supplementary Table S1. List of antibodies used for Western blotting and immunohistochemistry.

Antibody	Clone	Host	Dilution	Manufacturer/Reference
Pan-Cytokeratin	polyclonal	rabbit	1:200	Abcam, Cambridge, UK
Vimentin	EPR3776	rabbit	1:500	Abcam, Cambridge, UK
Phospho-p44/42 MAPK (ERK1/2)	Thr202/Thr204	rabbit	1:2,000	Cell Signaling Technology, Danvers, MA, USA
P44/42 MAPK (ERK1/2)	3A7	mouse	1:2,000	Cell Signaling Technology, Danvers, MA, USA
Vinculin	E1E9V	rabbit	1:2,000	Cell Signaling Technology, Danvers, MA, USA
Peroxidase AffiniPure anti-Rabbit IgG (H+L)		goat	1:10,000	Jackson ImmunoResearch Europe Ltd, Cambridgeshire, UK
Peroxidase AffiniPure Goat Anti- Mouse IgG (H+L)		goat	1:10,000	Jackson ImmunoResearch Europe Ltd, Cambridgeshire, UK
IRDye® 800CW anti-rabbit IgG sAb*		goat	1:15,000	LI-COR Biosciences, Lincoln, NE, USA
IRDye® 680LT anti mouse IgG sAb*		goat	1:15,000	LI-COR Biosciences, Lincoln, NE, USA
MmuPV1 L1/L2	polyclonal	rabbit	1:4,000	Eurogentec, Seraing, Belgium
Phospho-Histone H2A.X (Ser 139)	20E3	rabbit	1:300	Cell Signaling Technology, Danvers, MA, USA
Cytokeratin 17	polyclonal	rabbit	1:1,500	Abcam, Cambridge, UK

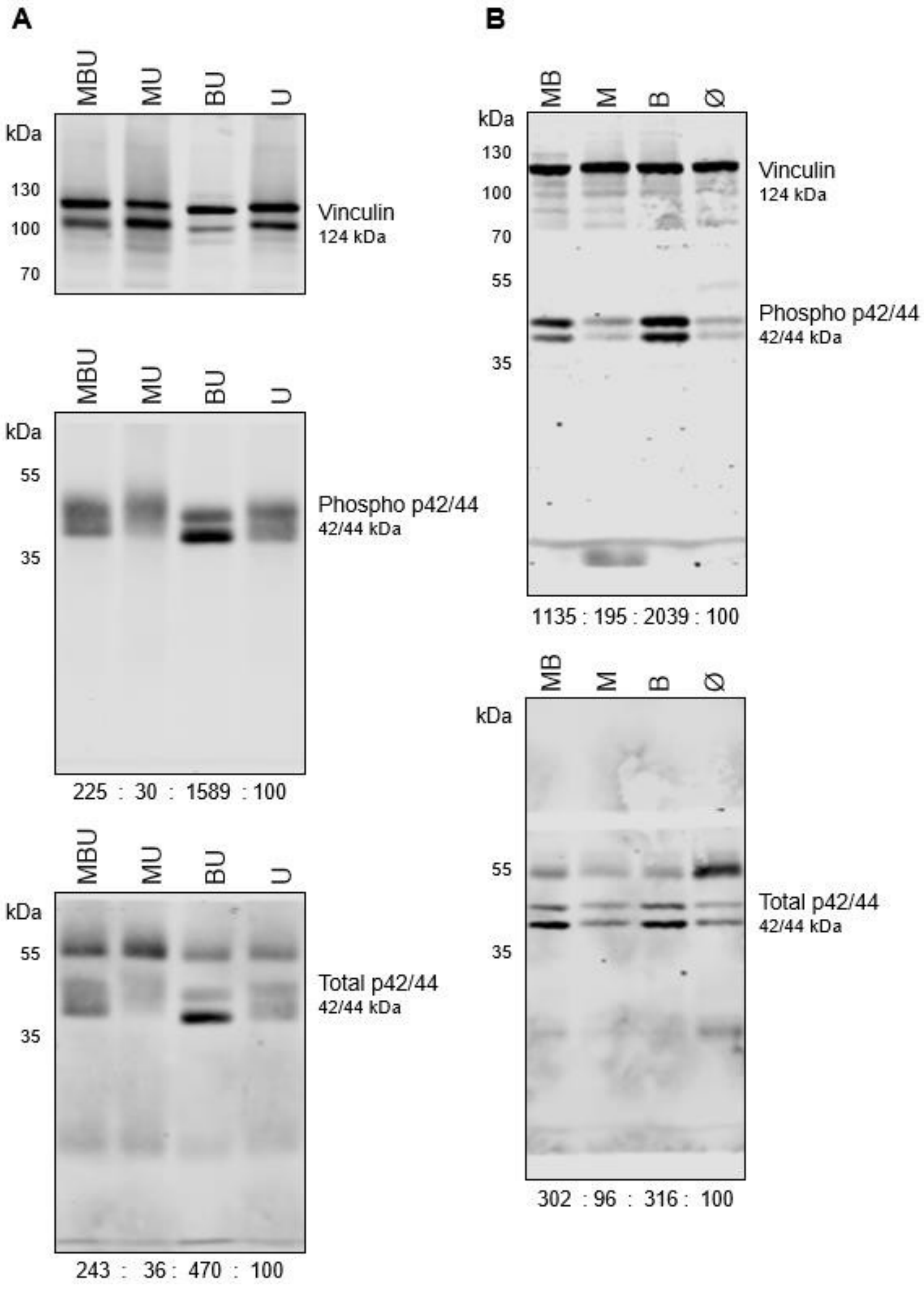

Supplementary Figure S 2. Uncropped Western blot of Figure 2 - Expression of phosphorylated (phospho) and total ERK1/2 in murine skin tissues. The intensity ratio of each band of interest is given below.
